# Modification of extraction method for community DNA isolation from salt affected compact wasteland soil samples

**DOI:** 10.1016/j.mex.2017.01.002

**Published:** 2017-01-17

**Authors:** Purvi Zaveri, Rushika Patel, Meghavi Patel, Devki Sarodia, Nasreen S. Munshi

**Affiliations:** Institute of Science, Nirma University, Sarkhej-Gandhinagar Highway, Ahmedabad 382 481, Gujarat, India

**Keywords:** Community DNA isolation from wasteland soil, Wasteland, Compact saline soil, Community DNA isolation, Modified enzymatic lysis method, Aluminium ammonium sulphate, Humic acid

## Abstract

To overcome the issue of interferences by salt and compactness in release of bacterial cell required for lysis, method described by Yeates et al. (1998), was optimized for isolation of genomic material (Deoxyribo Nucleic Acid, DNA) from soil microbial community by addition of Al(NH_4_)SO_4_. Very low total viable count was observed in the samples tested and hence use of higher amount of soil is required primarily for DNA isolation from wasteland soils. The method proves itself efficient where commercially available bead beating and enzymatic lysis methods could not give isolation of any amount of community genomic DNA due to compact nature and salt concentrations present in soil.

• The protocol was found efficient for soil samples with high clay content for microbial community DNA extraction.
• Variation in lysis incubation and amount of soil may help with soil samples containing low microbial population.
• Addition of Al(NH_4_)SO_4_ is crucial step in humic acid removal from extracted DNA samples for soil samples containing high salinity and clay particles.

## Graphical abstract

**Figure:**
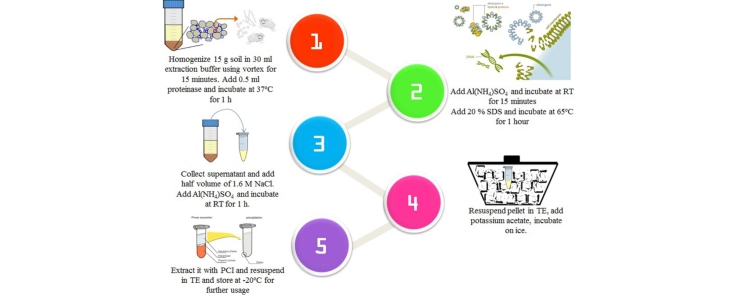

## Method details

**Fig. 1:**
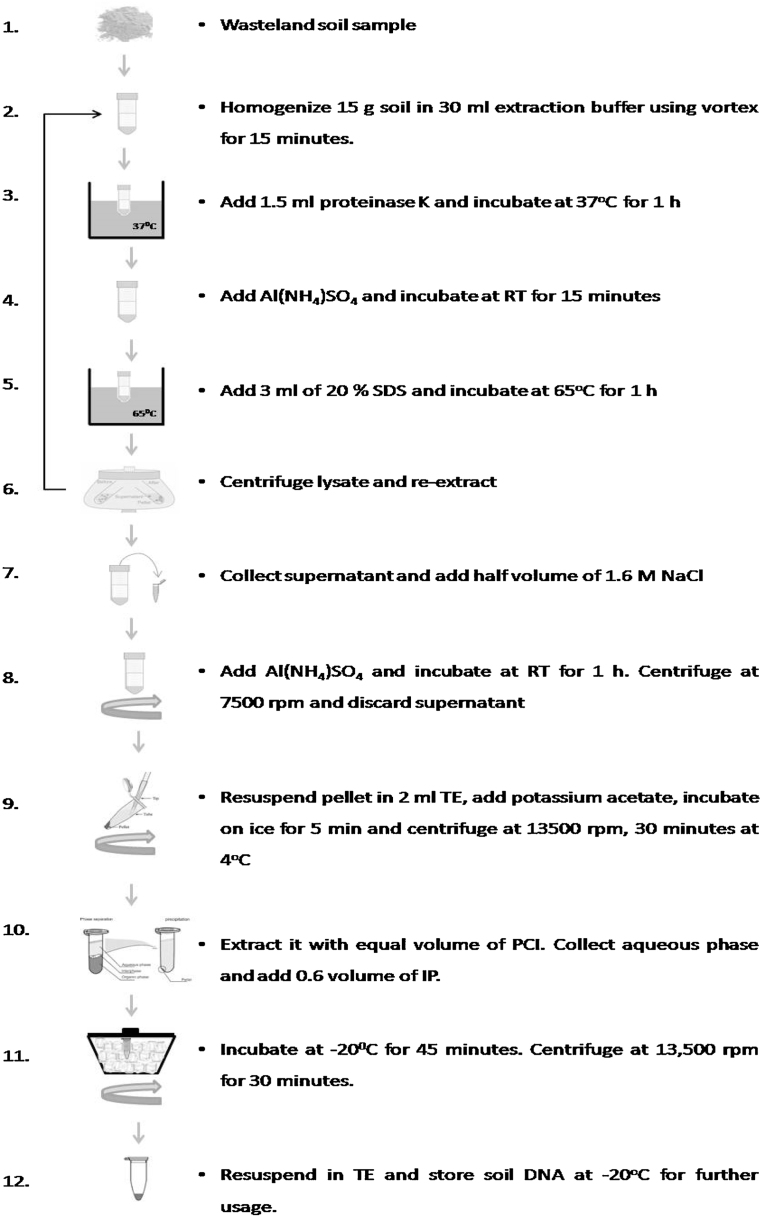
Workflow of microbial community DNA isolation for salt affected wasteland soil.

Method described by [1] for community DNA isolation from various types of soil samples was modified for wasteland soil samples collected from coastal areas of Gujarat. Extremely low microbial population ( <10^1^ cfu/g of soil) was detected and hence higher amount of sample was processed for microbial community DNA isolation. For preparation of the solutions and glassware used, sterile Deoxyribonuclease (DNase) and Ribonuclease (RNase) free water was used as and when required. Commercially available kit (bead beating) and enzymatic lysis methods tested without any modification could not yield any DNA from such type of soil sample. Community DNA isolation method involving use of hexadecyl-trimethylammonium bromide (CTAB) even could not successfully extract DNA from wasteland soil [2]. Bench modifications were performed only for enzymatic lysis method and are described in this paper. The flow chart of working protocol is described in Fig. 1. The process optimized is as follows.

### Soil sample preparation (Steps 1–2)

Soil samples were collected from Dholera, Bhavanagar, Gujarat, India (22.248°N 72.195°E). Collected soil samples were dried overnight and homogenized properly to give uniform mixture. Fifteen g (dry weight) soil was mixed with 30 ml extraction buffer (100 mM Tris-HCl, 100 mM Sodium EDTA (Ethylene Diamine Tetra Acetic acid) and 1.5 M NaCl [pH 8.0]) (final volume: ∼40 to 45 ml). The contents were vortexed for 1 min. Specific characteristics of soil like texture and physicochemical features are presented in Table 1 [3]. The organic carbon content in wasteland soil was found to be less than 1% (0.6 ± 0.2%). The salt content in soil ranged between 2 and 4%. Volume of the soil sample was changed due to the low microbial population found on Plate count agar (log cfu/g ranges between 1.29 to 5.06 ± 0.34 during summer and post monsoon season respectively), however in case of increasing the soil amount from 15 to 50 g led to huge humic acid contamination and pure DNA could not be extracted. The purpose was to obtain DNA concentration which is visible on agarose gel electrophoresis and usable for further amplifications.

**Table 1:** Physicochemical parameters of soil sample.

Parameter	Virgin soil	Wasteland soil
pH	8.4 ± 0.2	9.3 ± 0.1 *
Electrical conductivity (dS m^−1^)	0.1 ± 0.01	3.2 ± 0.5**
Water holding capacity (%)	43 ± 4	61 ± 3 *
Moisture content (%)	1.6 ± 0.9	12 ± 0.9 **
Sand (%)	80 ± 4	53.5 ± 1.38
Clay (%)	12.5 ± 2	37 ± 8 *
Silt (%)	1.9 ± 0.5	9.7 ± 0.7**

Note: ‘*’ = *P* value < 0.05; ‘**’ = *P* value < 0.01; when compared to virgin soil, n = 3.

The physicochemical characteristics were significantly different for wasteland soil as compared to virgin soil. High compactness in wasteland soil can be attributed to high silt and clay content in this soil (46.7%) as compared to that in virgin soil (14.4%). Reduction in cell lysis efficiency was found to correlate with higher clay content of soils as mentioned by [4].

### Cell lysis (Steps 3–6)

Proteinase K (1.5 ml) (Thermofisher, India) was added to samples prepared in step 1–2 and they were incubated at 37 °C for 1 h. The content was mixed intermittently for proper distribution during incubation. One molar **Al(NH_4_)SO_4_** (∼3.5 to 5.0 ml) was added to the total volume of mixture to achieve the final concentration of 100 mM and tubes were incubated for 15 min at 30 ± 2 °C temperature. SDS was added (3 ml; 20%) and tubes were incubated at 65 °C for 1 h. Al(NH_4_)SO_4_ was added to mixture for removal of humic acid and salt interferences. No further increase in the DNA concentration was observed with prolonged incubation after 1 h thus, it was kept up to 1 h only. Samples were centrifuged at 7500 rpm for 10 min at 30 ± 2°C temperature and supernatant was collected (∼30 to 35 ml). Soil pellet was re-extracted again with 10 ml extraction buffer by resuspending soil pellet and step 3–6 as shown in Fig. 1 were repeated [5]. During second extraction, 1 ml SDS (20%) was added due to proportionate reduction in starting volume of extraction buffer.

### Removal of cellular protein and PCR contaminants (Steps 7–8)

After discarding soil pellets, supernatants collected from both extractions (∼ 40 to 45 ml) were mixed and distributed in two halves, which were transferred to two 50 ml centrifuge tubes and were added with half volume of 1.6 M NaCl (10 to 11.5 ml) and 1 M **Al(NH_4_)SO_4_** was added to obtain final concentration of 100 mM (∼3 ml was required). Tubes were incubated at room temperature (30 ± 2°C) for 1 h and centrifuged at 7500 rpm for 20 min. The concentration of Al(NH_4_)SO_4_ obtained after step II as mentioned above is 100 mM and step III is adding another 100 mM of Al(NH_4_)SO_4_. Final concentration of Al(NH_4_)SO_4_ is required to be 200 mM for efficient removal of humic acid. The change in the addition of reagent may drastically affect DNA isolation.

### DNA precipitation (Steps 9–11)

Supernatant was discarded and pellet was resuspended in 2 ml TE buffer (Tris EDTA buffer: 10 mM Tris-HCl [pH 8.0], 1 mM Sodium EDTA [pH 8.0]) and were equally distributed in four 2 ml capacity centrifuge tubes. Potassium acetate (7.5 M) was added to achieve final concentration of 0.5 M (∼0.03 ml was required). Samples were placed in ice for 45 min and centrifuged at 13,500 rpm for 30 min at 4 °C. Higher concentration of potassium acetate and longer centrifugation time was required as compared to the method mentioned by [6]. The reported protocol [6] refers to centrifugation at room temperature; we modified it for the protection of fragile DNA pellet during further extraction steps.

The aqueous phase was extracted with equal volume of Phenol:Chloroform:Isoamyl alcohol (25:24:1) and DNA was precipitated by adding 0.6 vol of IP (∼0.6 ml). Samples were incubated at −20 °C for 45 min and centrifuged at 13,500 rpm for 30 min at 4 °C.

### Electrophoresis and visualization of extracted DNA (Step 12)

**Fig. 2:**
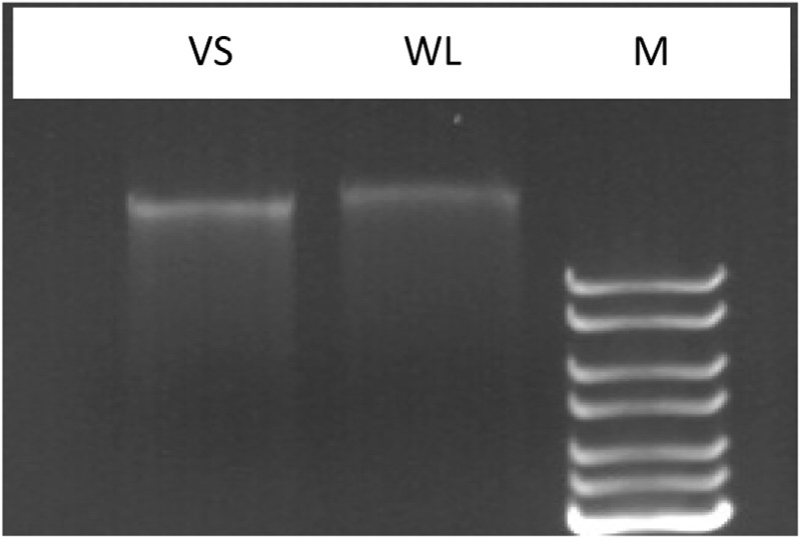
Agarose gel electrophoresis of soil community DNA isolated after aluminium hydroxide addition. (Community DNA from VS: Virgin Soil, WL: Wasteland Soil and M is Maker DNA with upper most band of 10 Kb.)

All the four pellets were dried at room temperature (30 ± 2 °C), re-suspended in 10 μl TE buffer individually and were pooled, electrophoresis was performed [7]. As displayed in Fig. 2, considerable amount of DNA was obtained from virgin as well as wasteland soil samples which were found to be suitable for further PCR reactions (data not presented).

The modified procedure could extract DNA from the wasteland soil samples too, however when processed for virgin soil the procedure did not yield high amount of DNA as expected. The fact should be considered that other standard protocols being used by various community DNA isolation kit could not help at all in extraction of DNA from wasteland soil samples.
